# The Mini-SIPS: development of a brief clinical structured interview guide to diagnosing DSM-5 Attenuated Psychosis Syndrome and training outcomes

**DOI:** 10.1186/s12888-022-04406-z

**Published:** 2022-12-13

**Authors:** Scott W. Woods, Cole Lympus, Thomas H. McGlashan, Barbara C. Walsh, Tyrone D. Cannon

**Affiliations:** 1 Department of Psychiatry, Yale University School of Medicine, New Haven, CT USA; 2 Department of Psychology, Yale University, New Haven, CT USA

**Keywords:** Clinical high risk (CHR) for psychosis, Diagnosis, Structured interview, Rater training

## Abstract

**Objective:**

The Mini-SIPS, a condensed version of the Structured Interview for Psychosis-Risk Syndromes (SIPS), is intended to efficiently identify for clinicians the minimum information needed to support a DSM-5 Attenuated Psychosis Syndrome (APS) diagnosis.

**Methods:**

The instrument and the DSM-5 criteria are accessible through the online training program.

**Results:**

Most individuals (67.5%) in the first 212 to complete the training program indicated an intended use of the Mini-SIPS exclusively for clinical purposes. Performance on the post-training quiz was excellent for those with and without prior training in structured diagnostic interviewing.

**Conclusion:**

The Mini-SIPS, and accompanying training program, are offered as public-domain clinical resources to the mental health community.

**Supplementary Information:**

The online version contains supplementary material available at 10.1186/s12888-022-04406-z.

## Introduction

Based on retrospective descriptions dating from the time of Bleuler [1], the last quarter century has witnessed concerted efforts to identify patients at risk for psychosis prospectively, beginning with the pioneering work of Yung and McGorry [2]. As would be expected when forecasting prospectively, not all patients identified as at risk develop psychosis: current meta-analytic estimates are 20% after 24 months [3]. Since prospective conversion to psychosis is not deterministic, the term “prodromal” is generally not used and several alternate designations are found in the literature including “clinical high risk” syndrome (CHR). Meta-analysis of epidemiology studies estimates the prevalence of these syndromes at 1.7% in the youth and young adult general population and 19.2% in the youth and young adult clinical population [4]. Recent evidence suggests that 88% of subsequent first episode patients experienced a retrospectively identifiable CHR syndrome before psychosis onset [5]. Research instruments used for prospective identification have included the Comprehensive Assessment of At-Risk Mental States (CAARMS) [6] and the Structured Interview for Psychosis-risk Syndromes (SIPS), with its imbedded Scale Of Psychosis-risk Symptoms (SOPS) [7].

**Fig. 1:**
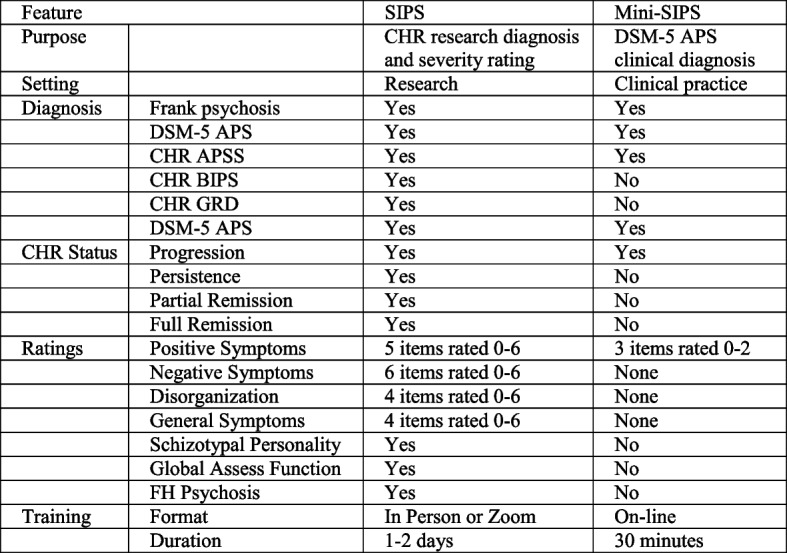
Comparison of SIPS and Mini-SIPS

Developed beginning in 1996, the SIPS has been employed in North America extensively and in Europe, Asia, Africa, and South America [8]. Reliability in the research setting has been excellent, with a median kappa across 16 reports of 0.89 [8]. Predictive validity for conversion to psychosis has also been strong, with rates in a meta-analysis seven times higher in referrals to SIPS evaluation who met criteria in comparison to referrals who did not [9]. Medians of published intra-class correlation reliability values for the SOPS rating scale have been excellent: 0.90 for the total score across nine studies, 0.88 for positive symptoms across 21 studies, 0.86 for negative symptoms across twelve studies, 0.80 for disorganization symptoms across eleven studies, and 0.88 for general symptoms across nine studies [8]. In addition to the SOPS symptom ratings, the SIPS also includes evaluations of global functioning, schizotypal personality, and family history of psychosis (Fig. 1). The instrument typically requires 1–2 h to administer, and rater training requires 1–2 days, administered in a group in-person format or over video conference. The SIPS lays out diagnostic criteria for the three CHR syndromes based on attenuated positive symptoms, brief intermittent psychosis, and genetic risk with functional decline originally described by Yung and McGorry. Since 2013 it has also included current status specifiers (progression, persistence, partial remission, and full remission) for each of the three syndromes [10]. The current version 5.6.1 [11] improved consistency of anchor wording across items and scale points.

Approximately ten years ago, the American Psychiatric Association DSM-5 Task Force and its Psychotic Disorders Workgroup engaged in an extensive evaluation of the reliability, validity, and utility of the CHR construct and elected to include the most common of the three CHR syndromes [12, 13] in the manual as a condition for further study, the Attenuated Psychosis Syndrome (APS) [14]. Criteria for DSM-5 APS are similar to those for SIPS Attenuated Positive Symptom Syndrome but also require both distress and impairment. As a condition for further study, DSM-5 APS was not allocated a unique diagnostic code; however, it was included as one of four examples of Other Specified Schizophrenia Spectrum and Other Psychotic Disorder (298.8 and ICD-10 F28). Subsequently, the US Substance Abuse and Mental Health Services Administration (SAMHSA) invested in clinical care for individuals at CHR at more than 20 sites [15], including a mix of community-based settings and academic medical centers. Overall structure of care fit with local practice but generally included a specialized CHR care team imbedded within a larger organization. SAMHSA initially required use of the SIPS for patient identification, and our trainer group [11] provided SIPS workshops for the SAMHSA CHR sites.

Shortly after the SAMHSA CHR clinics began operation, our group began receiving requests for a shorter version of the SIPS specifically designed for clinical rather than research use. Clinics were finding that 1–2 h was a lengthy period for diagnostic assessment, especially when third-party payors would often not reimburse for the full time required. In addition, some of the ratings recorded, while useful for characterizing CHR subjects in research studies, were not essential for making the clinical diagnosis. Moreover, staff turnover was unfortunately not uncommon at some of the clinics, and the extensive training needed for new hires posed resource challenges. We acknowledged that research-level precision or breadth of assessment was often not required in the clinical context and agreed to work on a briefer version for clinical use and a brief on-line training program, described here. We also report characteristics of the first two hundred individuals who completed the training program. The Mini-SIPS instrument is available online [16].

## Methods

### Evaluation of the SIPS

The first step was to judge which elements of the SIPS were minimally required for the clinical diagnostic task: to distinguish patients at CHR from those who were already psychotic and from those who were neither at CHR nor psychotic. Even though the negative, disorganization, and general symptom ratings are useful patient descriptors, only the attenuated positive symptom ratings are required for a diagnosis of CHR or for a SIPS diagnosis of psychotic disorder. It was also apparent that the 0–6 rating scale in the original SIPS version of these items, while helpful for grading symptom severity in longitudinal research within CHR individuals, could be simplified if only clinical diagnosis were of interest. Moreover, three of the five SIPS positive symptom ratings (unusual thought content, suspiciousness, grandiosity) mapped onto different subtypes of attenuated delusions, allowing further simplification into a single delusion-like symptoms item. Lastly, since many of the SAMHSA clinics were using the DSM-5 Other Specified Schizophrenia Spectrum and Other Psychotic Disorder (298.8) and associated ICD-10 (F28) codes for billing purposes, ratings for the two less common CHR syndromes not contributing to a DSM-5 APS diagnosis were not essential.

### Elimination of features not essential for clinical DSM-5 APS diagnosis

Features included in the SIPS and Mini-SIPS are shown in Fig. 1. Based on the analysis above we determined that the Mini-SIPS would not address negative, disorganization, or general symptom ratings. The 7-point SIPS attenuated positive symptoms severity scales were condensed to three points (0 = within normal limits, 1 = present at attenuated severity, 2 = frankly psychotic). The five SIPS positive symptom ratings were reduced to three (delusions, hallucinations, and disorganized communication). Global functioning, schizotypal personality, and family history rating, which are not required for the DSM-5 APS diagnosis, were also not included.

One feature of the SIPS, the verbatim query questions relating to attenuated positive symptoms, were deemed essential to detecting symptoms that might otherwise go undiscussed without the structured queries.

### On-line training

The Mini-SIPS training program was designed by the authors of the study and deployed online using the Yale University Qualtrics Program, a University-supported website and licensed version of the Qualtrics Research Suite. An institutional review board of the Yale Human Research Protection Program (FWA00002571) determined that the research and its inclusion of an on-line participant consent as part of the training program were exempt under 45 CFR 46.104 (2)(ii) and ethics approval was therefore waived. All methods were carried out in accordance with relevant guidelines and regulations in the Declaration of Helsinki—Ethics approval and consent to participate section. The research group advised mental health professionals about the availability of the training program by sending out emails via listservs and mentioning it during talks at scientific conferences and SAMHSA clinic coordination meetings. Participation in training was voluntary, unpaid, and otherwise unsolicited.

## Results

### Mini-SIPS clinical structured interview

Based on the considerations described above, we reduced the 48-page SIPS 5.6.1 research version to a four-page clinical version (the Mini-SIPS). Page 1 offers rating instructions at a level of detail consistent with the more limited aims of the clinical instrument. Page 2 retains all of the attenuated positive symptom queries from the SIPS. Page 3 permits rating the three DSM-5 positive symptom types on the restricted severity range and includes checkboxes for recording based on the interview as to whether sufficiently severe symptoms meet the additional requirements of the DSM-5 criteria. Page 4 guides frank psychosis and DSM-5 APS diagnoses based on the symptom and functioning ratings.

### Mini-SIPS training

All participants provided written informed consent. The training program indicates that individuals should have read the DSM-5 criteria for APS and have prior experience in psychiatric diagnostic interviewing. Participation in the online program is anonymous; however, participants are asked questions about their educational background, current profession, work setting, and demographics. The training program discusses the importance of focusing on positive symptoms and incorporating other factors when considering an APS diagnosis, such as being at least 12 years of age, a minimum IQ of 70, and considering presence of severe neurological disorder or traumatic brain injury that may complicate diagnosis.

The online training program reflects the procedure detailed on page 1 of the Mini-SIPS, while also providing some additional tips for conducting the interview. The online program proceeds to describe the three qualifying symptoms and the range of possible symptom ratings. Guidance is also provided for other considerations, such as ruling out those with frank psychosis and documenting whether an APS diagnosis is suggested or not.

Once participants consent and view the training materials, they proceed to the quiz, the final step in the program. The quiz is comprised of 14 questions and includes vignettes of all three symptoms at each of the three severity ratings as well as questions regarding diagnostic criteria and interviewing approaches. Feedback is immediately provided to all participants after each question is answered. The feedback includes their answer, the correct answer, an explanation, and the original question in case participants want to review it again. Once the quiz is complete, participants are asked if they want to be sent a record of completion of this training course. If so, they provide an email address at which to receive an electronic certificate.

### Training program results

**Fig. 2:**
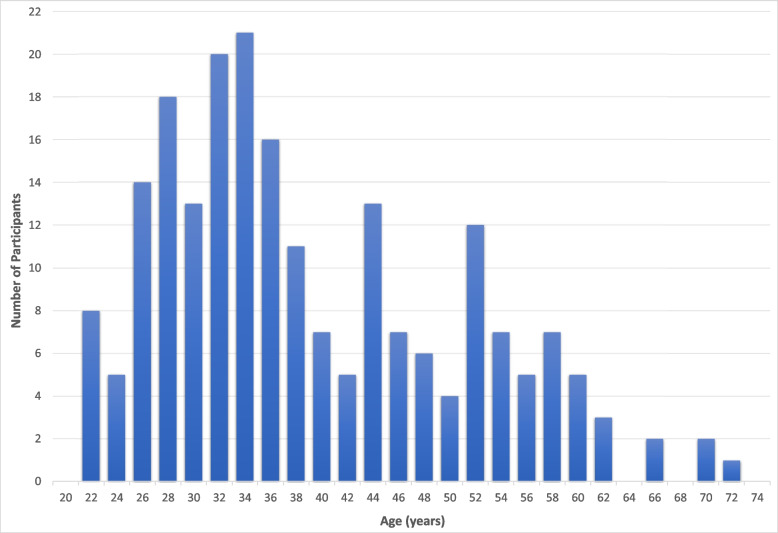
Age distribution of training participants

We aggregated data for the first 212 participants who completed the training program. All participants reported English as their primary language, with 82.5% identifying as female and the others identifying as male (16%) or non-binary (1.4%). The majority of the participants identified as white and not Hispanic or Latino (85.4%, Table 1). Figure 2 presents the age distribution of the participants (*M* = 39.59, *SD* = 11.54). The participants varied somewhat in their intended use of the training, largely participating in the training for clinical practice (67.5%) compared to those who intended to use the training for research purposes or both clinical and research purposes (10.8% and 27.7% respectively). Most of the participants (72.2%) work in a community-based setting (e.g., community mental health center). The remaining participants came from academic settings. As shown in Table 2, a wide variety of health-related professionals participated in the training. Relatedly, Table 3 shows the variability in the educational degrees of participants. Furthermore, 55.7% of the participants indicated that they had received training in structured interviewing for psychiatric diagnoses (e.g., SCID, SADS, Kiddie-SADS), and 61.3% reported having received training in structured interviewing for clinical high risk for psychosis syndromes (e.g., SIPS, CAARMS). Of note, some participants (7.5%) indicated they had previously completed the Mini-SIPS training, implying this was at least their second time attempting the training program. Due to the anonymous design of the study, we were unable to track which individuals were completing the Mini-SIPS training program for a second time or more.

**Table 1:** Racial distribution of training participants

Race	*n*	Percentage
American Indian or Alaska Native	1	0.5%
Asian or Asian American	18	8.5%
Black or African American	17	8.0%
Interracial	14	6.6%
White	162	76.4%
Total	212	100%

**Table 2:** Professional backgrounds of training participants

Profession	*n*	Percentage
Non-Mental Health Professional	2	0.9%
Nurse	4	1.9%
Occupational Therapist	7	3.3%
Other Mental Health Professional	83	39.2%
Other Physician (e.g., General Medicine)	2	0.9%
Psychiatrist	15	7.1%
Psychologist	27	12.7%
Researcher	25	11.8%
Social Worker	47	22.2%
Total	212	100%

**Table 3:** Highest educational degrees of training participants

Degree	*n*	Percentage
BA or BS	19	9.0%
MA	53	25.0%
MD	15	7.1%
MD and PhD	1	0.5%
MSW	45	21.2%
None	4	1.9%
Other doctoral degree	9	4.2%
Other master’s degree	32	15.1%
PhD	26	12.3%
PsyD	8	3.8%
Total	212	100%

Participants differed greatly in how much time they took to complete the training program (*Mdn* = 54.02 min). Only 30.2% of participants completed the training assessment in 30 min or less, the target duration for the program. However, a majority (52.8%) of participants completed in an hour or less, and 75% of participants completed in 2 h or less. Given that the training program is on-line and self-paced, it is possible that some participants paused the training program at one or more points; such “breaks” are included in the total completion time.

Participants performed well on the post-training assessment in terms of percent accuracy across the 14 quiz questions (*M* = 87.07, *SD* = 10.74). A Welch’s two sample t test showed that those who had training in structured interviewing for psychiatric diagnoses (e.g., SCID, SADS, Kiddie-SADS) received a higher post-assessment score (*M* = 89.0) than those that did not have such training (*M* = 84.7, *t* (183.49) = -2.92, *p* = 0.004). Those who had training in structured interviewing for psychiatric diagnoses were more likely to work in an academic setting (37.3%) as compared to those that did not have such training (16.0%, chi-square = 11.9, df = 1, *p* < 0.001), but were similarly likely to have received master’s or doctoral degrees (86.4%) as compared to those that did not have such training (92.6%, chi-square = 2.0, df = 1, *p* = 0.155). Furthermore, those who had received training in structured interviewing for clinical high risk for psychosis syndromes (e.g., SIPS, CAARMS) trended toward a significantly higher score (*M* = 88.0) than those who did not have such training (*M* = 85.5, *t* (183.65) = -1.68, *p* = 0.09). Those who had received training in the SIPS or CAARMS were similarly likely to work in an academic setting (30.8%) as compared to those that did not have such training (23.2%, chi-square = 1.4, df = 1, *p* = 0.229) and were also similarly likely to have received master’s degrees or higher (90.8%) as compared to those that did not have such training (86.6%, chi-square = 2.02, df = 1, *p* = 0.155).

## Discussion

The chief advantages of the Mini-SIPS compared with existing instruments commonly used in research settings are significantly reduced times for interview completion as well as for rater training. Based on the 87% average rate of correct responses in the post-training assessment, the training appears relatively effective despite its brevity. These features may help to remove obstacles to the establishment of CHR clinical programs and services that potentially could lead to a reduction in the incidence of psychosis. Based on the characteristics of the first two hundred individuals who completed the training program, it appears that the demand is reasonably high for a streamlined instrument for the clinical diagnosis of APS in community settings.

### Training time

One of the main intentions in designing the Mini-SIPS training course was to create a tool that required a short time to complete. Only 30% of participants, however, completed the training assessment in 30 min or less. Our initial interpretation was that the training took longer in practice than expected; however, other interpretations should be considered as well. Participants may have spent time reviewing the DSM-5 APS after logging onto the program rather than before logging in as we had anticipated. Moreover, on reviewing the distribution of the training times, which was strongly skewed toward longer times, it seems likely that the flexibility of allowing participants to use as much time as they needed to complete the program could have allowed other factors to influence the amount of time people used, such as external distractions, choosing to finish the program in divided time portions, forgetting they had not fully completed the study training, etc. The maximum time one participant used to complete the study was > 10,000 min, or nearly 7 days. In any case, that more than half of participants completed in less than an hour represents a substantially smaller training burden than the 1–2 days required for the SIPS research instrument.

### Intended use

Since the Mini-SIPS is intended to be a clinical instrument, we were surprised that slightly more than 10% of participants indicated that they intended to use the Mini-SIPS solely for research purposes. While we of course cannot control how professionals use an instrument that is in the public domain, we emphasize that most research applications would benefit from the additional information contained in, and the substantial reliability and validity [8] associated with, the research instrument. Similarly, some clinical practices may value features of the SIPS that could not be included in the Mini-SIPS (Fig. 1) and so prefer to continue to use the SIPS when time and resources permit. Features of the SIPS not included in the Mini-SIPS that could lead to such a preference include the SIPS rating scale for monitoring outcomes and the provision for determining less common CHR syndromes not reflected in DSM-5 Attenuated Psychosis Syndrome.

### Characteristics of training participants

As shown in Fig. 2, the modal training participant was in their early thirties. This age distribution is consistent with anecdotes we hear from community-based clinics where more recently hired staff can tend to be more likely to participate in training programs.

### DSM-5 Attenuated Psychosis Syndrome

Recently there has been interest in revisiting the possible placement of APS as a uniquely-coded diagnosis in the DSM-5.1 [17]. Consideration for placement there would require evidence for the inter-rater reliability of the APS clinical diagnosis beyond that provided by the limited sample studied in the DSM-5 field trials [18]. Of course, the clinical diagnostic reliability of any condition would be influenced by how familiar the studied clinicians were with the condition, whether they received training and how much, and whether a fully unstructured clinical diagnostic method or a semi-structured clinical instrument were used. Future field trials of APS might consider employing the Mini-SIPS and its available on-line training. An additional design could potentially compare Mini-SIPS APS diagnoses from clinicians with full SIPS APS research diagnosis in the same patients.

### Strengths and limitations

A strength of this study is that the training participants seem reasonably representative of mental health clinicians in community-based practice in terms of race (Table 1), profession (Table 2), and education (Table 3). A limitation, however, is that there is a significant imbalance in the gender representation of the participants who completed the training: more than 80% self-identified as female. We did not seek specifically to advise female clinicians about the availability of the instrument and do not have an explanation for the gender imbalance.

A limitation for the employment of the Mini-SIPS in current practice is that the clinical reliability studies with the Mini-SIPS and diagnostic studies evaluating agreement between SIPS research diagnoses and Mini-SIPS clinical diagnoses mentioned above have not yet been conducted. Given that the Mini-SIPS was developed from the full SIPS by removing features not essential for the clinical diagnosis while retaining the same diagnostic concepts and much of the same wording, the excellent inter-rater reliability record of the SIPS in the research setting suggests that future clinical reliability studies with the Mini-SIPS are likely to find at least acceptable reliability. Clinicians, however, should be aware of this limitation when considering use of the Mini-SIPS as a guide to DSM-5 APS clinical diagnosis.

Recently, SIPS authors have been collaborating with authors of another CHR diagnostic interview, the Comprehensive Assessment of At Risk Mental States (CAARMS), to harmonize the two research instruments where possible and to generate the two instruments’ differing sets of CHR criteria from the same research interview. This effort is expected to have relatively little impact on DSM-5 APS diagnosis guided by the Mini-SIPS, but when it has been fully completed and validated we will consider whether to issue a revised version of the Mini-SIPS incorporating minor modifications.

Finally, our current method employing anonymous on-line training is both a strength and a limitation. The strength is that the ease of use for trainees promotes dissemination which in turn we hope will promote public health. The limitation is that we cannot enforce our recommendation that trainees have prior experience in psychiatric diagnostic interviewing. This limitation imposes a burden on clinics who may employ staff without such prior experience to provide supervision and oversight. We stress that the Mini-SIPS by itself does not establish a psychiatric diagnosis; it is a guide or tool that assists in the diagnostic process. The responsibility for the ultimate diagnostic determination in clinical practice lies not with the tool but with the appropriately-credentialed practitioner.

## Summary

The Mini-SIPS and accompanying training program are offered as public-domain resources to the mental health community. The brief duration of the Mini-SIPS guide to diagnostic assessment may improve the bottom line for clinics serving patients with DSM-5 APS and permit resources to be more focused on treatment. The availability of the training program as an on-line resource may facilitate awareness of the DSM-5 APS diagnosis and enable community mental health sites to maintain a trained staff despite staff turnover.

## Supplementary Information

**Additional file 1: Supplementary Materials 1. **Mini-SIPS Training Program data _April 27 2021.

## Data Availability

All data generated or analyzed during this study are included in this published article (and its Supplementary Materials file 1).
